# Acceptability of single screening and treatment policy for the control of malaria in pregnancy: perceptions of providers and pregnant women from selected health facilities in Lindi region, Tanzania

**DOI:** 10.1186/s12936-021-03782-3

**Published:** 2021-06-08

**Authors:** Chonge Kitojo, Frank Chacky, Emmanuel S. Kigadye, Joseph P. Mugasa, Abdallah Lusasi, Ally Mohamed, Erik J. Reaves, Julie R. Gutman, Deus S. Ishengoma

**Affiliations:** 1 US President’s Malaria Initiative, United States Agency for International Development, Dar es Salaam, United Republic of Tanzania; 2 The Open University of Tanzania, Dar es Salaam, United Republic of Tanzania; 3 National Malaria Control Programme, Dodoma, United Republic of Tanzania; 4 PMI Impact Malaria Population Services International (PSI), Dar es Salaam, United Republic of Tanzania; 5 US President’s Malaria Initiative, Centers for Disease Control and Prevention, Dar es Salaam, United Republic of Tanzania; 6 Malaria Branch, Division of Parasitic Diseases and Malaria, Center for Global Health, Centers for Disease Control and Prevention, Atlanta, GA USA; 7 National Institute for Medical Research, Dar es Salaam, United Republic of Tanzania; 8 Faculty of Pharmaceutical Sciences, Monash University, Melbourne, Australia; 9 School of Public Health, Harvard University, Boston, MA USA

**Keywords:** Malaria in pregnancy, Single screening and treatment, Acceptability of SST, Malaria, Tanzania

## Abstract

**Background:**

Tanzania started implementing single screening and treatment (SST) for all pregnant women attending their first antenatal care (ANC) visits in 2014, using malaria rapid diagnostic tests (RDTs) and treating those who test positive according to the national guidelines. However, there is a paucity of data to show the acceptability of SST to both pregnant women and health care workers (HCWs), taking into consideration the shortage of workers and the added burden of this policy to the health system. This study assessed the perceptions and opinions of health service users and providers to determine the acceptability of SST policy.

**Methods:**

Pregnant women and HCWs in eight health facilities in two districts of Lindi region (Kilwa and Lindi) were interviewed using semi-structured questionnaires with open and close-ended questions. Both qualitative and quantitative data were collected, including demographic characteristics, women’s experience, their perception on SST and challenges they face when receiving services for malaria offered at ANC. Experience of HCWs regarding the implementation of SST as part of routine services and the challenges encountered when providing ANC services for malaria in pregnancy (MIP) were also assessed.

**Results:**

Of the 143 pregnant women interviewed, 97% viewed testing favourably and would wish to be tested for malaria again, while 95% were satisfied with services and reasons for testing during the first ANC visit. Nearly all (99%) would recommend their fellow pregnant women to be tested for malaria and all women recommended that the Ministry of Health should continue the SST strategy. This was despite the fact that 76% of the women experienced pain and 16% had anxiety as a result of finger prick. Sixteen HCWs (mostly nurses) were interviewed; they also viewed SST implementation favourably and reported feeling empowered to use RDTs for malaria screening. The main challenge identified by HCWs was that nurses are not allowed to prescribe anti-malarials to women who test positive and need to refer them to the outpatient department for treatment.

**Conclusion:**

SST was considered an acceptable approach to control MIP by HCWs and pregnant women, and they recommended the continuation of the policy. In addition, consideration should be given to implementing a task-shifting policy to allow nurses to dispense anti-malarials to pregnant women.

## Background

Malaria in pregnancy (MIP) is a major global public health problem, with approximately 12 million pregnant women exposed to malaria and 822,000 low birth weight infants in 33 high/moderate malaria transmission settings in African countries in 2019 [1]. MIP is associated with an increased risk of maternal and fetal complications, including maternal anaemia, spontaneous abortion, low birth weight (LBW; < 2500 g), and infant and maternal death [2–5]. In Tanzania, over 93% of the population lives in areas where malaria is transmitted, and it is estimated that about 1.7 million pregnant women are at risk of getting malaria. The burden of malaria is significantly higher in areas around Lake Victoria and Southern parts of Tanzania with persistently high transmission intensity [6]. However, recent reports show that the country-wide prevalence of malaria in pregnancy in Tanzania declined from 8.1% in 2014 to 6.7% in 2017[7]. This decline is attributed to the scale-up of malaria interventions which have been implemented by the National Malaria Control Programme in the past two decades [7, 8].

Tanzania implements the World Health Organization (WHO) recommended package of interventions to control MIP, including providing pregnant women with intermittent preventive therapy during pregnancy (IPTp) using sulfadoxine-pyrimethamine (SP), insecticide treated nets (ITNs) at first antenatal clinic (ANC) visits, and effective malaria case management with prompt diagnosis and treatment with effective anti-malarials [9]. Specifically, IPTp using at least three doses of SP from the second trimester is recommended for the control of MIP [10], but its effectiveness might be compromised in areas with high SP resistance such as East and Southern Africa including most parts of Tanzania [11].

In addition to these interventions, since 2014, Tanzania has implemented screening of all pregnant women with malaria rapid diagnostic tests (RDTs) at the first ANC visit and treating those who are positive according to the national guidelines [9]. This approach, referred to as single screening and treatment (SST), contributes to clinical management of the women, ensuring that they promptly receive an effective anti-malarial if infected, which is particularly important given the extremely high level of SP resistance detected in some areas of Tanzania, such as Tanga (prevalence of *Pfdhps*-581G reaching 56.6%) [12]. The SST policy is implemented nationwide in all 184 councils, and testing rate has increased from 36·7% in 2014 to 88.6% in 2017 [7]. A recent study conducted in Lindi region revealed that SST might be useful for identifying and treating asymptomatic women from high and moderate malaria transmission areas leading to effective case management and improved control of MIP [13]. In addition, data from SST can be useful in the surveillance of malaria especially in regions with low transmission where national surveys cannot detect parasitaemia due to limited sample size [7]. The ANC data can also be useful for establishing the burden of malaria at macro and micro-geographic levels, allowing stratification of interventions [8].

For successful implementation and utilization of new health services/interventions such as SST, health service users and providers’ perceptions and opinions are among the key factors/determinants [14]. Several studies were conducted in Ghana and Indonesia to investigate the perception and acceptability of malaria interventions such as intermittent screening and treatment (IST) *versus* IPTp and SST, respectively [15–18]. The findings of these and other studies [18–21] showed that although women experienced discomfort with a finger prick, they expressed more positive sentiments towards IST than IPTp because of the value of malaria diagnosis and preference of an artemisinin-based combination therapy (ACT) compared to SP, due to side effects caused by SP[16, 20, 21]. However, treatment adherence was an issue for women with asymptomatic malaria because most of the asymptomatic patients thought that they did not need to be screened and treated [19]. In addition, other studies showed that healthcare workers (HCWs) accepted the screening and treatment of pregnant women because of the value of identifying asymptomatic pregnant women and providing treatment [13, 21], which may reduce the chance of women becoming infectious reservoirs [22]. In addition, HCWs reported that RDTs were simpler and faster to administer than microscopy [21].

Although previous studies showed high acceptability and utilization of MIP interventions by pregnant women and HCWs, some of these studies were conducted in trial settings (the interventions were provided under strict study conditions) that translated into better effectiveness of the interventions, such results might not be attained in routine health facility settings. Thus, it is important to assess the acceptability of SST to both pregnant women and HCWs outside the trial settings, to fully understand the burden imposed by the SST strategy on an already over-stretched health system in Tanzania, as introduction of RDTs in ANC may be perceived as adding to the workload [15]. This study presents the perceptions and experiences of women and HCWs for determining the acceptability of SST in routine ANC settings.

## Methods

### Study design

This was a cross-sectional exploratory study of pregnant women attending their first ANC visits and HCWs involved in providing ANC services conducted between October and December 2018. The data obtained included demographic characteristics, knowledge of MIP, and experience of providers and women regarding the use of RDTs at first ANC visits.

### Study sites

The study was carried out in eight health facilities in Kilwa and Lindi districts councils (DCs), in Lindi region, southern Tanzania. These districts are made up of rural and peri-urban areas, where malaria transmission is moderate, with bimodal rainfall peaking in November and April [6]. The prevalence of malaria in Lindi region was 15% and 12% among children aged 0–59 months in the national household surveys conducted in 2016 and 2017, respectively [23, 24], and ranged from 13 to 20% among pregnant women between 2014 and 2017 [7].

Lindi region was purposively selected from the list of Southern zone regions with parasite prevalence among under-fives of > 12% [23]. Kilwa and Lindi districts were randomly selected from among the five districts in Lindi region as previously described [13]. In each district, four health centres were then purposively selected from a sampling frame of all 11 health centres in the two districts (73%) based on high volume of ANC attendance, with a minimum of 9 pregnant women per month in Lindi district and 29 women in Kilwa district. The populations of the two districts were relatively similar, 190,744 and 194,143 in Kilwa and Lindi, respectively [25].

### ANC services

Tanzania adopted the WHO recommendation on focused ANC (FANC) in 2002. FANC was intended to align important services and care that promote early detection of infections and initiation of early and appropriate treatment to prevent subsequent complications. Testing at first ANC includes syphilis, malaria, and HIV. Preventive measures are also emphasized, including intermittent preventive treatment of malaria in pregnancy (IPTp), insecticide-treated bed nets, tetanus toxoid immunization, and iron and folic acid for prevention of anaemia [26]. Tanzania has recently adopted the 2016 WHO recommendation of increasing ANC contacts from four to eight [27].

### Study population and sample size

All pregnant women attending their first ANC in the eight selected facilities who were tested for malaria on the day the study team visited the facility or had been tested during the same pregnancy were eligible. Those who consented to participate were interviewed using a structured questionnaire.

### Health care workers

A minimum of two HCWs working at the ANC of each facility were recruited to participate in in-depth interviews. In facilities with more than two HCWs, two respondents were selected based on availability on the day of interview, providing written consent, and experience with SST implementation. Respondents included Enrolled Nurses (EN), Assistant nursing officers (ANO), and Nursing Assistants (NA) who were directly involved in the provision of ANC services, including testing for malaria at the first ANC visit.

### Data collection

To determine the perception and experience of pregnant women and HCWs regarding SST policy, qualitative and quantitative approaches involving a standardized questionnaire and in-depth interviews were used to collect data from respondents. The questionnaire, which was designed in English and translated into Kiswahili, included both open and close-ended questions. Data collectors were oriented on the use of the questionnaire for women and the in-depth interview checklist with HCWs. For the pregnant women, the questionnaire consisted of demographic characteristics, an assessment of knowledge and perceptions about ANC and FANC, MIP and SST. It also covered women’s experience with malaria testing at first ANC visit, challenges encountered when receiving ANC services, and whether respondents would advise their fellow women to be tested for malaria. For HCWs, a checklist was used for in-depth interviews which included questions on MIP, and experiences, benefits, challenges, and recommendations for future implementation of SST. Data collection was supervised by the principal investigator and co–investigators. During supervision, they checked completeness of the responses and other things, including review of 10% of questionnaires to check for completeness and accuracy of information, to ensure quality data were collected. No personal identifiers were used; all participants were given study specific identifiers.

### Data management and analysis

#### Quantitative data

The data were entered using an entry template into an SPSS Version 20 software (IBM, Corporate, NY, USA) and subsequently cleaned before analysis was performed using the same software. Both descriptive analysis (frequency and percentage) and cross-tabulations were performed. Statistical significance was assessed with chi-square test or t-test as appropriate; a *p*-value < 0.05 was considered statistically significant.

#### Qualitative data

The data were analysed to determine perceptions and experience of HCWs using the feasibility framework developed by Bowen et al*.* and adopted by Hill et al*.* [21, 28], describing eight areas to be considered when conducting feasibility studies. This module was adapted and used for data analysis and presentation of results. Common ideas representing the following six relevant themes/areas were selected from the interview transcripts: acceptability, demand, implementation, practicality, integration, and adaptation. Therefore, the following key areas were assessed: (1) to what extent do pregnant women and HCWs accept the SST strategy? (2) Any changes already made or need to be made to the routine system to ensure effective implementation of the SST policy (adaptations)? and (3) any recommendations to ensure effective implementation of the SST policy. In addition, women and HCWs’ knowledge of MIP and SST policy, and experience and perceptions with implementation of SST were included in the analysis. More details on definitions and key outcomes are shown in Tables 3 and 4.

## Results

### Pregnant women

#### Baseline characteristics of women

A total of 143 pregnant women were interviewed at eight health facilities; 63% were from Kilwa DC. The largest proportion of women (46%) were aged 14–25 years old while 41% were aged 26–35 years; 82% were married and 59% were housewives. Sixty-nine percent of the women had only primary school education, 48% were multigravidae, and only 36% were in the first trimester (gestational age less than 12 weeks). In both districts, 95% of the women had received a bed-net at their first ANC visits and 93% slept under a net a night before their visit to the clinic. Almost all women (99%) reported having been tested for malaria on the day of the interview. Overall, 14% (n = 20) of the women were positive by RDTs, with no significant differences between Kilwa and Lindi DCs (13 vs 15%, respectively, *p* = 0.58) (Table 1). Among the health facilities, positivity rates ranged from 5% at Nanjilinji, Pangaboi, and Masoko to 40% at Tingi health centre.

**Table 1:** Characteristics of pregnant women interviewed during their first ANC visits in Kilwa and Lindi DCs

Variable	Kilwa (n = 90)	Lindi (n = 53)	Total (n = 143)
Age			
14–25	44 (49%)	22 (42%)	66 (46%)
26–35	35 (39%)	23 (43%)	58 (41%)
36–45	11 (12%)	7 (13%)	18 (13%)
46 and above	0 (0%)	1 (2%)	1 (1%)
Gestational age			
< 12wks	37 (41%)	14 (26%)	51 (36%)
> 12wks	53 (59%)	39 (74%)	92 (64%)
Gravidity			
Primigravidae	19 (21%)	16 (30%)	35 (24%)
Secundigravidae	24 (27%)	14 (26%)	38 (27%)
Multigravidae	47 (52%)	25 (47%)	70 (48%)
Level of education			
Did not attend school	20 (22%)	6 (11%)	26 (18%)
Primary	56 (62%)	43 (81%)	99 (69%)
Secondary and above	14 (16%)	4 (8%)	18 (12%)
Employment status			
Housewife	49 (54%)	36 (68%)	85 (59%)
Employed in formal sector	2 (2%)	0 (0%)	2 (2%)
Entrepreneur /small business	6 (7%)	2 (4%)	8 (6%)
Farmer	33 (37%)	15 (28%)	48 (34%)
Marital status			
Married	81 (90%)	37 (70%)	118 (82%)
Single	2 (2%)	2 (4%)	4 (3%)
Widow	7 (8%)	14 (26%)	21 (15%)
Use of bed-net			
Pregnant women slept under a bed-net last night	84 (93%)	49 (92%)	133 (93%)
Pregnant women received a bed-net during 1^st^ visit	86 (96%)	50 (94%)	136 (95%)
Testing for malaria at first ANC visit			
Tested with RDT	89 (99%)	52 (98%)	141 (99%)
RDT positive	12 (13%)	8 (15%)	20 (14%)

### Knowledge and experience of women about ANC and MIP

Of the pregnant women, 95% (n = 136) were aware of the importance of attending ANC during pregnancy, and around three-quarters of women (76%, n = 109) identified 12 weeks as the appropriate time to start ANC. Of the respondents, 88% (n = 127) had heard of MIP and ways to prevent malaria during pregnancy. Most of the women (72%, n = 104) had heard of MIP prevention messages at ANC clinics. When asked about the most effective method of preventing MIP, most (52%, n = 75) women reported ITNs, 34% (n = 49) mentioned IPTp, while only 5% (n = 7) mentioned screening and treatment of malaria at ANC.

### Women’s experience of malaria testing at the first ANC

Nearly all women (92%, n = 132) reported experiencing discomfort with malaria testing at the first ANC visit (blood for all necessary testing, including malaria, was obtained from a single finger prick); 76% experienced pains and 16% had anxiety as a result of finger prick. Despite the discomfort, 97% of the women would wish to be tested for malaria again and 95% were satisfied with the services provided and the reasons for testing during the first ANC visit. Of the women, 99% would recommend to their fellow pregnant women to be tested for malaria, and all women recommended that the Ministry of Health should continue the SST strategy because women appreciated knowing whether or not they are infected with malaria. In addition, 39% of the pregnant women requested more information on the prevention of malaria during pregnancy and 43% on the appropriate time to test for malaria, as pregnant women wondered why they were tested while they were not sick.

### Characteristics of health care workers

Sixteen service providers from the eight health centres were interviewed; most of them (94%) were female and all but one HCW were trained on the use of RDTs. The single untrained nurse was newly employed by the government. With the exception of the newly hired nurse, most of the HCWs (75%) had at least 5 years’ experience; two (13%) had more than 20 years’ experience. All HCWs were involved in performing RDTs but none prescribed and/or dispensed anti-malarials.

### Health care workers’ perception and experience with testing using RDTs

The majority of HCWs had positive perceptions of SST implementation as part of routine ANC services. They appreciated how the policy helped to identify asymptomatic women who would have gone undetected in the absence of screening, endangering the mother and unborn baby (Table 2).

**Table 2:** Summary of key findings: acceptability and demand of SST from the perspective of healthcare providers

Health care workers felt that it is important to test in order to identify women with malaria and treat them immediately. “*if we leave women with malaria undetected, they might experience complications during their pregnancy. Some women are coming with no symptoms so if not tested, they will walk around with malaria hence endanger themselves and their babies*”, HCW in Lindi DC
Overall, HCWs had good knowledge of the SST policy and acknowledged having been trained in performing RDTs as reported by one respondent; “*I understood this is a national policy that every woman has to be tested for malaria when attending the first ANC visit*”, HCW in Lindi DC
Regarding training, one respondent asserted that; “*Most of us were oriented on the national policy and trained on use of RTDs for diagnosis of malaria at the first ANC visit*”, HCW in Kilwa DC Most the HCWs recommended that the policy should continue following the benefits mentioned above. However, they recommended more training, especially for newly employed personnel. “*I think this policy should continue as it has benefits in identifying women with malaria and enabling doctors to treat them*”, HCW in Kilwa DC
The burden to HCWs was assessed; most of the respondents felt that it was not a burden because the test for malaria is integrated into the routine laboratory tests already being performed within routine ANC services. The same nurses who perform other tests, such as HIV, are also performing RDTs and mostly using the same finger prick “*We will conduct a test anyway because we have to do HIV and syphilis tests. Therefore, the same blood can be used to test for malaria, so it is not an additional task*”, HCW in Kilwa DC. In addition, the HMIS has been revised and testing data are collected and reported in the DHIS2. HCWs are required to record the SST results in the routine ANC registers

SST implementation was observed in all eight facilities and HCWs were knowledgeable, felt empowered, and were able to perform the testing for malaria without any problems. Commodities were available on the day of interview; no HCW reported stock-outs of malaria commodities, such as ACT and RDTs in the past 6 months, with the exception of quinine, which the majority of facilities reported had been out of stock in the preceding 6 months (Table 3).

**Table 3:** Summary of key findings: implementation, practicality and adaptation of SST

Implementation	SST was being correctly implemented in all 8 health centres visited- and 99% of the women attending 1^st^ ANC were tested for malaria, and those who were positive received treatment; Nurses expressed that RDTs were easy to perform compared to microscopy. “*RDTs can be performed by any nurse without much experience and expertise; and most of us have been trained on the use of RDTs. I feel it is easy and quick to use compared to microscopy. I can test my patients with no problem*”, ANC Nurse, Kilwa DC
RDTs availability	None of the facilities in the study area had experienced any stock-outs in the past year. The ANC used only RDTs and not microscopy for malaria testing. Ordering of additional tests was completed on time, and facilities performed quantification based on consumption. Redistribution of RDTs was happening within the districts using a group text service, as reported by a respondent. “*We are using group texts *via* an electronic platform which has been created at the district level where we communicate to each other to share stock-out information and this helps us in redistribution of commodities*”, ANC nurse, Kilwa DC
Anti-malarial availability	ACTs were available in all 8 health centres. No HCW reported stock-out of ACTs in the preceding 6 months. On the other hand, most facilities had experienced periodic stock-outs of quinine tablets in the past 1 year. “*We have not experienced a stock-out of malaria commodities in our facility. Usually we do our orders on time and we receive the orders according to our requests. The only commodity that has been a challenge is Quinine*”, HCW from Lindi DC
Anti-malarial prescription challenge	Anti-malarials were prescribed by clinicians at OPDs. Women with positive RDT results had to be escorted to OPD for treatment to avoid queues at OPDs and also to make sure no women went home without treatment. Pregnant women with a negative RDT were given IPTp, provided that they were in their second or third trimester. Women with a negative RDT who were symptomatic were referred to the clinician for further assessment and were treated per the Ministry of Health guidelines for symptoms or any other problems which were detected. None of the nurses prescribed anti-malarials. All treatment information was kept/recorded at the outpatient department (OPD). “*We only conduct RDT for pregnant women who are coming for their first ANC visits. For those who are positive, we usually escort them to the clinicians for further assessment and are given anti-malarials. Most of us working here at ANC do not prescribe or dispense any anti-malarials to pregnant women*. *Escorting pregnant women to OPD is sometimes challenging especially when we have many clients to attend to and as you know we are not many who work here but we do our best to help our clients*”, HCW from Lindi DC

The majority of HCWs felt comfortable with implementation of the SST, because it is already integrated into the routine ANC services. Blood tests, such as syphilis and HIV, are routinely conducted by ANC nurses as part of FANC at the first ANC visit [26], and the RDT was integrated into the system such that it did not require an additional blood draw. In addition, the reporting is already integrated into the Ministry of Health’s Health Management Information System (HMIS).

The main challenge reported by the HCWs with regard to implementation of SST was that ANC nurses were not allowed to prescribe anti-malarials for women who tested positive. Instead, the nurses needed to escort pregnant women to the outpatient department (OPD) for treatment. This was considered the main challenge, and a potential factor that could increase waiting time for the women possibly affecting other ANC services given the shortage of staff (Table 3). Service providers recommended that SST should be continued, but that frequent supervision and on-the-job training of HCWs, particularly new staff, should be prioritized (Table 4).

**Table 4:** Summary of key findings: recommendations of HCW for effective implementation as part of integration and adaptation of the SST

Recommendations from HCWs	All HCWs recommended SST implementation to be continued but provided recommendations for improved implementation of SST. Refresher training and training for the newly recruited staff were recommended by one respondent: “*I think training of new staff is important for malaria testing at ANC and this will ensure nurses are doing their job well*. *In our facility, we received training from NMCP and Boresha Afya, but refresher training will be important as a reminder on testing procedures”*, HCW from Lindi DC Regular supervision was also highlighted by the HCWs as an important aspect of the SST implementation. “*When technical experts from NMCP and the council health management team (CHMT) come to supervise us, we learn new things and also, they point out problems that we could have not seen*”, HCW from Kilwa DC

## Discussion

Tanzania has been implementing the SST policy since 2014 [9] and the data collected from ANC has proved to be useful in improving case management of pregnant women and also in the estimation of malaria burden at micro-geographic levels [7, 29]. This study assessed the acceptability of SST in the selected health facilities in Lindi region, and provides information within the routine ANC services that will be used by the NMCP to strengthen delivery of the SST strategy. This is a unique advantage of these findings as other studies were done outside routine services. The findings of this study showed that SST services were available in all studied health facilities; and its implementation was viewed by women and HCWs as adding value to ANC attendance, with perceived health benefits for pregnant women and their babies. This is very important for the continuation of the SST policy. Since its launching in 2014, SST has been rolled out across the mainland Tanzania, with about 160,000 women being tested each month at ANC throughout the country [7]. Testing and reporting rates have improved significantly since 2014, reaching 88.6% and 98% by the end of 2017, respectively [7]. Availability of the services and acceptability are crucial for the effective implementation of the SST policy and utilization of the services by pregnant women [20].

### Acceptability and demand of SST

The findings showed that SST was acceptable to both pregnant women and HCWs, despite the discomfort and anxiety reported by most of the pregnant women who were tested for malaria. This suggests that women are willing to endure the discomfort of an RDT in the process of confirming if they have malaria or not, to enable them to receive appropriate treatment that will protect them and their unborn children from the consequences of malaria. These findings are similar to those of other studies which evaluated the acceptability of malaria interventions, including intermittent screening and treatment (IST) [16, 20]. These studies showed that acceptability plays an important role in the individuals’ adherence and compliance to malaria interventions. Further, IST and SST studies showed that women accepted a finger prick for malaria testing despite the pain it caused [16, 18]. In addition, if the results are well communicated, it might help to build trust between the clients and HCWs. It may also provide an opportunity for the women to discuss and consider uptake of other health interventions against MIP and other diseases. The positive attitude of HCWs and willingness to provide service has the potential to enhance coverage and ensure sustainability of SST and other services, which will benefit mothers and their unborn babies.

HCWs appreciated that the SST policy identified women with asymptomatic infection, who would otherwise have gone undetected. A study conducted in the same health facilities to evaluate the SST policy showed that about 60% of women had asymptomatic infections, suggesting that this policy may have a substantial impact especially in the women who are in the first trimester when IPTp is contraindicated [13]. This is particularly important in areas of high SP resistance like Tanzania. In addition to being at high risk for poor outcomes, pregnant women with asymptomatic parasitaemia comprise an infectious reservoir, which could propagate transmission [23]. This underscores the need for early identification and treatment of infections to reduce sequelae of malaria in pregnancy and accelerate the ongoing elimination efforts [30]. While pregnant women accepted SST, about 95% reported not being aware of SST prior to their first ANC. Women requested more information on malaria testing and the reasons for testing. This signified a positive interest in SST but also highlighted the need to improve the information provided to women regarding the benefits of testing, particularly for those without symptoms of malaria [30].

### Implementation and practicability

The majority of HCWs had a positive attitude towards implementation of SST as part of the national policy. Testing for malaria at ANC is already integrated within testing of other diseases, such as HIV and syphilis, as part of routine antenatal services. This is advantageous to the HCWs as it eliminates conducting multiple tests, saving time and reducing workload [21]. The existing integration of point of care tests has allowed HCWs to provide the results and treatment quickly, saving clients’ time as services are provided under one roof. Furthermore, HCWs perceived RDTs to be easy and quick to use in addition to reducing waiting time compared to microscopy. Ghanaian HCWs similarly had no concerns with incorporating RDT screening into their existing workload, as it is easy to use and they reported improved engagement with clients as a result of being able to provide them malaria testing results [31]. Regular supervision was highlighted by the HCWs as an important aspect of the SST implementation to improve malaria services including quality control and quality assurance (QAQC) for testing and treatment. Since Tanzania has a robust quality improvement initiative through the Malaria Services and Data Quality Improvement tool (MSDQI) [32], the proposed supervision and retraining can be potentially strengthened through the current system.

Supplies and commodities are central to effective and efficient implementation of SST. A major facilitator reported by HCWs leading to the successful implementation of SST was the continuous availability of malaria commodities such as RDTs and ACT. This is in line with the findings from a study conducted in Kenya which showed that the most important factor associated with malaria testing of pregnant women was the availability of diagnostics at the point of care [33]. Furthermore, interventions that increase the availability of malaria diagnostic services might also improve malaria case management in pregnant women [33]. On the other hand, a lack of commodities, such as RDTs, may have a negative impact on the implementation of SST; low testing rates have been largely attributed to stock-outs of RDTs [34]. Such stock-outs may be due to poor quantification and forecasting by health facilities [34]. Lessons learnt from this study show that availability of malaria commodities in Kilwa and Lindi districts was a result of better quantification and ordering practices, effective communication among the facilities, and the opportunity for redistribution of malaria commodities [13].

### Adaptation

Use of technology was highlighted by HCWs as an important element for maintaining stocks of malaria commodities. Evidence showed that digital health technologies can be harnessed to support access to medicines and pharmaceutical services. In this study, health facility staff used digital applications, such as group texts, to communicate and share information among them. The information helped to understand stock status at their facilities and those in nearby facilities. This information was used to redistribute RDTs and ACT to prevent stock-outs in facilities which had limited supplies. This underscores the need to build effective systems for triangulation of data from commodities, geo mapping, and other parameters of the pharmaceutical system and developing dashboards that will systematically provide early warnings to mitigate stock-outs and wastage of medicines and commodities [35].

The major challenge for effective implementation of SST, as reported by HCWs, was that nurses were not allowed to prescribe anti-malarials, thus, all pregnant women who tested positive had to be referred and escorted to the OPD for treatment. During busy clinics this can present a major challenge, as there may not be any other available nurse to attend to the remaining pregnant women. A similar issue was raised by a study in Indonesia where midwives at village level were not allowed to prescribe anti-malarials, and all positive pregnant women had to be referred to the health centre for treatment [21]. Task shifting policies allowing more efficient use of the available human resources [36] and increasing the efficiency of health care delivery should be considered [37]. This could be accomplished by allowing ANC nurses to dispense anti-malarials, particularly for women without other symptoms, as this is something which community health workers, who have less training than nurses, are able to do [21, 33].

### Recommendations

1. *Commodity supplies*: Without consistent supplies of MIP commodities, all other program efforts to increase coverage of MIP interventions will be rendered ineffective. Therefore, there is a need for the NMCP, through the Council Health Management Teams (CHMTs), to continue strengthening the coordination and supply chain efforts through imparting skills and knowledge in quantifying, forecasting, ordering, and distributing MIP commodities where there are shortages. This includes use of technology as demonstrated in this study where digital applications were used to share information on stock status for efficient re-stocking and/or re-distribution of RDTs and ACT medicines.
2. *SST awareness*: Most of the pregnant women were not aware of SST prior to their first ANC visit and expressed willingness to learn more about the test. This underscores the need to create awareness about SST given that about 60% are asymptomatic cases. In order to ensure effective SST implementation, health education needs to be strengthened to ensure that women understand the rationale for testing, the consequences of malaria and MIP, and the high rates of asymptomatic malaria with possible effects on the mothers and unborn children.
3. *Supportive supervision*: To ensure successful implementation of SST, regular supportive supervision from both regional/council and national levels (from NMCP) was deemed important by HCWs. They viewed this as an important opportunity for learning and providing/receiving feedback on their performance. Supportive supervision has been shown to help in identifying challenges, which can then be addressed through mentorship and on-the-job training.
4. *Training:* On-the-job training of HCWs was also emphasized as an opportunity to impart skills to new staff and a reminder on testing procedures. This was also viewed as a motivation to staff and an opportunity to learn new skills as well as improving performance of HCWs.

## Conclusion

SST was considered an acceptable approach for the control of MIP by both HCWs and pregnant women. The majority of pregnant women had a positive perception of SST, were willing to be tested and recommended to other women to be tested too. Pregnant women and HCWs supported continuation of the SST policy. However, consideration should be given to providing additional training on malaria testing for the current and new HCWs, in addition to effective supportive supervision and mentorship. An uninterrupted supply chain is important for the success; the use of technology reported in this study should be strengthened because it was useful and effective in the redistribution of MIP commodities among facilities and in maintaining the stock of RDTs and anti-malarials for ANC. Finally, consideration should be given to implementing a task-shifting policy to enable nurses to dispense anti-malarials to pregnant women.

## Data Availability

Data from this study can be accessed upon request through the corresponding author who will submit a formal request to the Ministry of Health.

## Abbreviations

ACTs: Artemisinin Combination Therapy
ANC: Antenatal Care
CHMT: Council Health Management Team
HCW: Health Care Worker
IPTp: Intermittent Preventive Treatment in pregnancy
ITNs: Insecticide-treated mosquito nets
LLIN: Long-lasting insecticidal nets
MIP: Malaria in Pregnancy
RDTs: Rapid Diagnostic Tests
NMCP: National Malaria Control Programme
NIMR: National Institute for Medical Research
OPD: Outpatient Department
SP: Sulfadoxine-Pyrimethamine
SST: Single Screening and Treatment
WHO: World Health Organization
